# Tuberculous Peritonitis

**Published:** 1899-12-30

**Authors:** 


### TUBERCULOUS PERITONITIS.

In the Harveian Lectures delivered before the Medical Society, Mr. Watson Cheyne points out that although in the present state of our knowledge the rationale of the cure which so often follows upon the performance of laparotomy in cases of tuberculous peritonitis is incapable of complete explanation the fact does not stand quite alone. There is a certain analogy between what happens in these cases and what takes place when we open an acute abscess, when, as is well known, if the operation is done at the proper time, pus ceases to form. Not only does cure seem to result in each case from mere opening of the cavity and evacuation of the morbid products, but the cases resemble each other in this also, that in both, if we operate too early, we fail to get the satisfactory result that follows if we intervene at the proper time, recurrence being apt to take place in both. The question of the abscess is as great a mystery as that of the tuberculous peritonitis. Putting on one side, however, all question of explanation, the fact remains that laparotomy in tuberculous peritonitis often leads to a real cure of the disease. This has been definitely proved, the abdomen having been opened for other reasons later on, when it has been found that not only has the tuberculous effusion disappeared, but that even the adhesions and the results of the tuberculous peritonitis have also gone. As to the time of operating, Mr. Watson Cheyne says that in practically all cases where improvement does not follow under medicinal treatment, say in from four to six weeks in acute cases, or in from four to six months in chronic cases, the abdomen should be opened, whether there be ascitic fluid or not. In most cases the operation is very simple. Where effusion is present without adhesions it simply consists in opening the abdomen in the middle line below the umbilicus, allowing the fluid to run out, aided by turning the patient on his side, and perhaps removing some of it by means of sponges, and then stitching up the wound again. Where adhesions are present care must be taken in opening the abdomen not to injure the intestine, and if the adhesions are firm it is better to leave them alone and close the wound rather than to try to force one's way in, unless fluid be present. In the dry, fibrous form, unless the intestine is becoming kinked or bound down, it is seldom advisable to attempt to separate the coils. If, however, obstruction is present it must be relieved, or an anastomosis must be made between the coils above and below. Finally, when pus is found it should be washed out by salt solution, and it is then well to introduce a little iodoform and glycerine emulsion into the cavity before closing it. There seems no advantage in draining. Better to thoroughly clean and then sew up. In doing this operation it will sometimes be found that the disease appears to spring largely from some primary focus, such as a tube or an appendix, and in such cases it becomes an important question whether such a primary focus should be removed. This depends much on the extent of the disease. If the whole peritoneum is affected then no such attempt should be made, but if the tuberculous peritonitis be limited to the immediate vicinity of the tube, or appendix and cæcum, the removal of the primary source is very important, and much better results are obtained by so doing than by simple laparotomy.

